# Intracoronary nicorandil induced hyperemia for physiological assessments in the coronary artery lesions

**DOI:** 10.3389/fcvm.2022.1023641

**Published:** 2022-11-02

**Authors:** Xia Yang, Qiang Yu, Junjie Yang, Jun Guo, Qinhua Jin

**Affiliations:** ^1^Department of Cardiology, The First Medical Center, Chinese PLA General Hospital, Beijing, China; ^2^Department of Hepatobiliary Surgery, The First Medical Center, Chinese PLA General Hospital, Beijing, China

**Keywords:** coronary artery disease, fractional flow reserve, nicorandil, ATP, pressure wire

## Abstract

**Objective:**

Maximal hyperemia is a key element of invasive physiological examination. The aim of this study was to investigate the efficacy and safety of intracoronary (IC) nicorandil in comparison with adenosine 5′-triphosphate (ATP) intravenous (i.v.) injection for fractional flow reserve (FFR) measurement in coronary artery lesions.

**Materials and methods:**

In this study, 46 patients who had their FFR measured were enrolled, including 51 lesions. Hyperemia was induced by bolus 2 mg nicorandil and ATP (40 mg ATP + 36 ml saline, weight × 10 ml/h) for FFR measurement. The safety and efficacy of IC nicorandil were evaluated.

**Results:**

The mean FFR values measured by nicorandil and ATP were 0.810 ± 0.013 and 0.799 ± 0.099, *p* < 0.001, respectively. There was a strong correlation between FFR measured by nicorandil and ATP (*r* = 0.983, *R*^2^ = 0.966, FFR_nicorandil_ = 0.937 × FFR_ATP_ + 0.061). The rate of FFR ≤ 0.75 in the nicorandil and ATP groups was 31.37 vs. 35.29%, respectively (*p* = 0.841), the consistency rate was 96.08%; the FFR ≤ 0.8 rate was 41.18 and 43.14%, respectively (*p* = 0.674), and the consistency rate was 90.20%. In five lesions, the FFR value measured by nicorandil ranged between 0.79 and 0.82, indicating inconsistency according to FFR ≤ 0.8. The blood pressure changes caused by nicorandil and ATP were 12.96 ± 6.83 and 22.22 ± 11.44 mmHg (*p* < 0.001); the heart rate changes were 2.43 ± 1.31 and 6.52 ± 2.87 beats/min, respectively (*p* < 0.001); and the PR interval changes were 6.0 (1.0–11.0) and 9.0 (2.0–19.0) ms, respectively (*p* < 0.001). Visual analog scale (VAS) scores in the nicorandil group were all in the range 0–2, while in the ATP group were mostly in the range of 3–5.

**Conclusion:**

Intracoronary bolus of nicorandil (2 mg) infusion induces stable hyperemia, and it could be considered as an alternative drug to ATP for FFR measurement with a lower side effect profile in most patients.

## Introduction

Coronary angiography has limitations in evaluating functional ischemia in coronary artery stenosis. In cardiac catheterization, fractional flow reserve (FFR) has been proven to be a reliable method for assessing the severity of coronary stenosis. Several clinical studies have shown that invasive coronary physiology examinations could improve patient outcomes and provide a more appropriate selection of patients who may benefit from percutaneous coronary interventions (PCI) (1–4). This has led to the recommendation of FFR in coronary artery revascularization guidelines (5–8). Maximal hyperemia is the crucial prerequisite to assess FFR correctly, and intravenous (i.v.) administration of adenosine or adenosine 5′-triphosphate (ATP) is still considered the gold standard.

Despite compelling evidence and recommendations from guidelines, the use of FFR is not frequent in the real world. In addition to its cost, some patients present contraindications to adenosine or ATP infusion. When an FFR examination is performed after adenosine or ATP infusion, more than 85% of patients experience discomfort, such as chest pain or dyspnea. In a few patients, adenosine or ATP infusion will cause severe side effects, such as atrioventricular block (AVB) or ventricular tachycardia, which may require the interruption of FFR assessment (9, 10).

In addition, some food and drink intake may affect the FFR value and the stability of ATP or adenosine. Recent studies mentioned that the consumption of caffeine, which is commonly consumed in 48 h before coronary angiography, may cause clinical data to be unbelievable (11).

Therefore, a more convenient and safer hyperemic agent may facilitate the use of FFR measurement in the cardiac catheterization laboratory.

Nicorandil (Sigmartw, Chugai Pharmaceutical, Japan), a coronary vasodilator that acts on both macro- and micro-vascular circulations, has been reported to be safe and cardio-protective when administered intracoronary (IC) in patients with coronary artery disease (CAD) (12). Some small studies showed that an IC bolus injection of nicorandil 2 mg is a simple, safe, and effective way to induce steady-state hyperemia for invasive physiological evaluations in patients undergoing angiography catheterization laboratory (13).

We performed this study to evaluate the feasibility and efficacy of IC nicorandil (Beijing SihuanKeBao Pharmaceutical Co. Ltd, China) compared with i.v. ATP infusion for FFR measurement in Chinese patients.

## Materials and methods

### Study population

Patients with angiographic epicardial coronary artery stenosis (50–90%) who agreed to have an FFR test were enrolled prospectively. Patients with acute myocardial infarction (within 7 days), regional wall motion abnormalities, reduced left ventricular systolic function [left ventricular ejection fraction (LVEF) < 40%], primary valvular or myocardial disease, severe liver insufficiency or renal dysfunction [estimated glomerular filtration rate (eGFR) < 30 ml/min], history of asthma/chronic obstructive pulmonary disease, and contraindication to ATP or nicorandil were excluded in this study. The angiographic exclusion criteria were left main (LM) lesion, right coronary artery (RCA) ostial lesion, and very distal lesion, which the pressure wire sensor could not cross. Patient’s clinical data, such as age, gender, diagnosis, CAD risk factors, LVEF, and medication utility histories were collected. The study protocol was approved by the institutional ethics committee board. All patients were properly informed prior to the procedure and gave their written consent to participate in the study. All patients must abandon caffeine 48 h after signing the consent willingly.

### Pressure measurements

All procedures were performed through the right radial artery using 6F guiding catheters without side holes. The guiding catheter and pressure-sensing guidewire are zero, respectively. After i.v. administration of heparin 100 IU/kg and IC nitroglycerin 200 mcg, a 0.014-inch pressure monitoring guidewire (St Jude Medical, Minneapolis, MN, USA) was calibrated and introduced into the guiding catheter. The pressure transducer was advanced just outside the tip of a 6 Fr guiding catheter, and the pressure measured by the sensor was then equalized (EQ) to that of the guiding catheter. If the EQ value is between 1 and ±9, the wire was then advanced distally to the target coronary stenosis. If the EQ value is not in the range of 1 to ±9, then pull back the wire and zero again till the EQ value is reasonable. Special attention was paid to avoid arterial pressure wave damping, unselective catheterization of the coronary ostia, and variation in the position of the pressure wire. FFR was calculated as the ratio of distal coronary pressure divided by aortic pressure obtained after the achievement of maximal hyperemia (14). The brachial vein was used for the systemic administration of ATP. An FFR value of ≤0.80 was considered the significant ischemic threshold. After measurement of the FFR value, pull back the pressure wire to just outside the tip of the guiding catheter. If the difference between catheter pressure and wire pressure is less than ±5 mmHg, then the FFR value was acceptable, otherwise, the FFR value should be measured repeatedly following the above steps. The time to the lowest FFR (time needed to reach >90% of the minimal value of Pd/Pa) was measured in both groups, and the plateau time (the time during FFR remained at >90% of its lowest value) was measured during the IC bolus of nicorandil.

After that, the wire was introduced distal to the stenosis and the baseline Pd/Pa was calculated (Pd: mean coronary pressure distal to coronary lesion and Pa: mean aortic pressure). After baseline Pd/Pa was recorded, the hyperemic efficacy of the following two successive methods was used: continuous i.v. ATP (40 mg ATP + 40 ml saline, weight kg × 10 ml/h, equate to 167 mcg/kg/min) and an IC bolus injection of nicorandil (2 mg). A continuous i.v. infusion of ATP was performed *via* a large forearm vein. To exclude the possible influence of the sequence of pharmacological agents, the i.v. infusion of ATP was followed by a nicorandil IC bolus in the first half of the patients, and vice versa in the second half. Each hyperemic stimulus was given after confirming that the Pa, Pd, and heart rate had recovered to their baseline values.

The visual analog scale (VAS) pain score was assessed during an i.v. infusion of ATP and an IC bolus of nicorandil (15). Using a 10 cm-length scale, measure the VAS score, 0 means no discomfort and 10 means the most severe and unbearable pain. Each patient selected one score after i.v. ATP and IC nicorandil, respectively. A 12-lead ECG was performed at baseline, during the FFR measurement. The PR interval was measured in Lead II by an independent cardiologist in a blinded fashion.

### Quantitative coronary angiography

The quantitative coronary angiography (QCA) was performed by an independent analyzer blinded to the results of the FFR. All images were obtained using a 6F guiding catheter after IC nitroglycerin 200 mcg. Each target lesion had at least two orthogonal views. The external diameter of the contrast-filled guide catheter was used as a calibration standard. Using an edge detection system (SiChuang QCA system, China), the minimal lumen diameter and the reference diameter were measured and the percentage diameter stenosis was calculated. The reference diameter was defined as the average value of the proximal and distal reference diameters.

### Statistical analysis

All statistical analyses were performed using STATA 11. Discrete variables were expressed as a frequency and a percentage and analyzed by Fisher’s exact test. Continuous variables were expressed as the mean standard deviation or median and interquartile range (IQR) (25–75th) and analyzed by the paired t-test or the non-parametric Wilcoxon test, as appropriate. Using the SK-test method to determine whether sample data are normally distributed or not. The relationship between the two groups was quantified with a coefficient of determination (*r* and *r*^2^) and regression analysis. A *p*-value of 0.05 (two-sided) was considered statistically significant.

## Results

### Patients’ clinical and angiographic characteristics

Between December 2020 and February 2021, 49 patients were screened. In total, 3 patients failed to enroll for the following reasons: incomplete pressure recording in one patient for severe atrioventricular block (AVB); guiding catheter pressure instability in one patient; and pressure drift in one patient. FFR comparisons were finally available in 46 patients with 51 lesions. The clinical and angiographic characteristics of study subjects are summarized in Table 1. The average age was 60.80 ± 8.83 years old. The majority of patients had been diagnosed with unstable angina, and 10.87% of patients had a previous PCI. The most target vessels were the left anterior descending artery (LAD) (84.31%), and the average angiographic percentage stenosis was 65.60 ± 10.03%.

**TABLE 1:** Patients’ clinical and angiographic characteristics.

Variables		Values
Age (years)		60.80 ± 8.83
Male [*n* (%)]		21 (45.65)
Hypertension [*n* (%)]		36 (78.26)
Hypercholesterolemia [*n* (%)]		8 (17.39)
Diabetes [*n* (%)]		18 (39.13)
Current smoker [*n* (%)]		10 (21.74)
Family history of CAD [*n* (%)]		5 (10.87)
Stable angina [*n* (%)]		15 (32.61)
Acute coronary syndrome	Unstable angina [*n* (%)]	25 (54.34)
	NSTEMI [*n* (%)]	4 (8.70)
	STEMI (>7 days) [*n* (%)]	2 (4.35)
LVEF [*n* (%)]		61.89 ± 3.85
Prior MI [*n* (%)]		3 (6.52)
Previous PCI [*n* (%)]		5 (10.87)
Medication [*n* (%)]	ASA	46 (100)
	Clopidogrel	46 (100)
	RAAS antagonist	26 (56.52)
	ß-Blockers	25 (54.35)
	CCBs	17 (36.96)
	Statins	41 (89.13)
Target vessel [*n* (%)]	LAD	43 (84.31)
	LCX	3 (5.88)
	RCA	5 (9.80)
Minimal lumen diameter (mm)		1.5 (1.2–2.0)
Reference vessel diameter (mm)		3.0 (2.6–3.3)
Percent diameter stenosis (%)		65.60 ± 10.03

Values are medians [interquartile range (IQR), 25th–75th] or *n* (%).

### Hemodynamic and electrocardiogram changes

The effects of two different hyperemia methods on blood pressure, heart rate, and heart conduction are shown in Table 2. An IC bolus of nicorandil produced fewer changes in the mean blood pressure, heart rate, and PR interval than an infusion of ATP (all *p*-values < 0.001). Transient AVB occurred in 2 (4.34%) patients with an infusion of ATP, and none with an IC bolus of nicorandil. Patients complained of more chest pain with ATP infusion than with nicorandil IC bolus (*p* < 0.001). VSA scores in the nicorandil group were 0–2, while in the ATP group, 89.13% of VSA scores were 3–5, and 4.34% were 6–8. About 97.5% of patients complained of chest discomfort.

**TABLE 2:** Hemodynamic changes and visual analog scale (VSA) pain score according to hyperemic agents.

	Nicorandil bolus	ATP infusion	*P*-value*
***n* = 51**			
Δ Mean blood pressure (mmHg)	12.96 ± 6.83	22.22 ± 11.44	<0.001
Δ Heart rate (/min)	2.43 ± 1.31	6.52 ± 2.87	<0.001
ΔPR interval (ms)	6.0 (1.0–11.0)	9.0 (2.0–19.0)	<0.001
***n* = 46**			
VAS pain score			<0.001
0–2	46 (100)	3 (6.52)	
3–5	0	41 (89.13)	
6–8	0	2 (4.35)	
9–10	0	0	

*Between nicorandil bolus and adenosine 5′-triphosphate (ATP) infusion.

### Hyperemic efficacy

**FIGURE 1:**
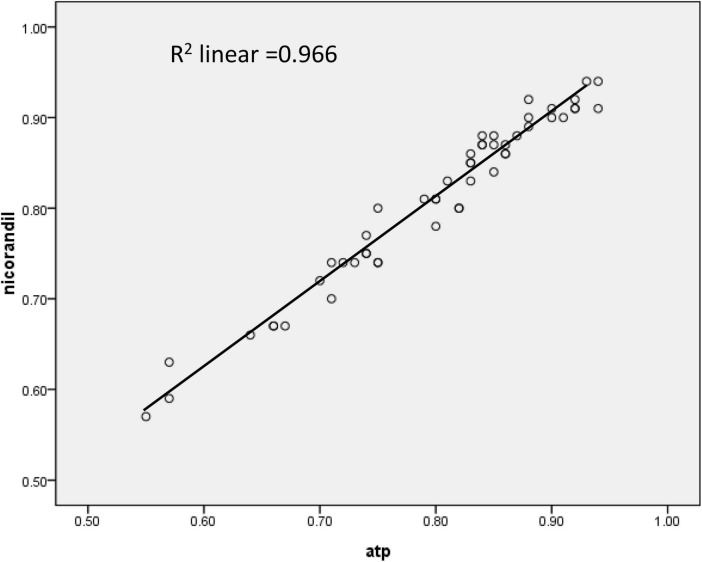
The correlation of fractional flow reserve (FFR) between an intracoronary nicorandil bolus (2 mg) and an intravenous infusion of adenosine 5′-triphosphate (ATP) (167 mcg/kg/min).

According to baseline Pd/Pa values, ATP, and nicorandil, all caused significant hyperemia and lowered hyperemic Pd/Pa values. FFR values with nicorandil 2 mg IC were commonly higher than those with ATP infusion (0.810 ± 0.013 vs. 0.799 ± 0.099, *p* < 0.001; Table 3). While a strong and linear correlation was observed between FFR with an IC bolus of 2 mg nicorandil and an infusion of ATP (*r* = 0.983, *R*^2^ = 0.966, *p* < 0.001; Figure 1). The time to the lowest FFR was shorter with an IC bolus of nicorandil 2 mg than with an infusion of ATP (13.24 ± 4.17 vs. 45.75 ± 5.25 s, *p* < 0.001), and the plateau time of an IC bolus of nicorandil was 25.4 s (15.6–32.8 s).

**TABLE 3:** Hyperemic efficacy and the number of functionally significant lesions according to two different hyperemia methods.

	Nicorandil bolus	ATP infusion	*P*-value*
FFR	0.810 ± 0.013	0.799 ± 0.099	<0.001
Time to the lowest FFR (s)	13.24 ± 4.17	45.75 ± 5.25	<0.001
Plateau time (s)	25.4 (15.6–32.8)	–	–
FFR ≤ 0.75 (*n*)	16 (31.37)	18 (35.29)	0.674
FFR ≤ 0.80 (*n*)	21 (41.18)	22 (43.14)	0.841

*Between nicorandil bolus and ATP infusion.

Functionally significant stenosis (FFR < 0.75) was seen in 16 (31.37%) patients with an IC bolus of nicorandil 2 mg and 18 (35.29%) patients with an infusion of ATP. The patients with FFR < 0.80 were 21 (41.18%) patients with an IC bolus of nicorandil 2 mg and 22 (43.14%) patients with an infusion of ATP.

The agreement rate of FFR ≤ 0.75 for an IC bolus of 2 mg nicorandil and an infusion of ATP was 96.08%; the agreement rate of FFR ≤ 0.8 was 90.20%, and there was a discrepancy in which the FFR value with nicorandil was between 0.79 and 0.82 in five lesions.

## Discussion

Fractional flow reserve is the most accurate method for discriminating which lesions are associated with ischemia in the catheterization laboratory, and current guidelines recommend its use when evidence of ischemia at non-invasive stress tests is not available (5–8). Nevertheless, the FFR is still underused in clinical practice. This is due to some practical reasons, such as the lack of understanding or belief in the FFR concept, the need to position a special wire with inferior handling characteristics, the need to make special connections, the lack of financial reimbursement, and the worry side effects of adenosine or ATP administration.

To avoid the disadvantage of adenosine or ATP, some studies have suggested the method without adenosine. In particular, Davies et al. proposed the instantaneous wave-free ratio (iFR) as a novel adenosine-free index of stenosis severity in the ADVISE study (16). The iFR is calculated by the ratio of the distal coronary artery pressure (Pd) to the aortic pressure in the diastolic period. The basic hypothesis of this technique is that there is a diastolic “wave-free” period when microvascular resistance is already constant and minimal and consequently does not need further vasodilation. However, the usefulness of this new tool is still debated and indeed presents several limitations. With or without hyperemia medication, microvascular resistance difference may exist, and iFR may underestimate the stenosis severity. This could explain, at least partially, the imperfect correlation between iFR and FFR observed in the ADVISE study. Therefore, a certain degree of hyperemia has to be provided to identify functionally significant stenosis correctly.

In China, it is difficult to acquire adenosine in clinics because it is not domestic and imported products are also very scarce. The price of ATP is cheaper than adenosine. Most cardiologists only use ATP as a hyperemia medication instead of adenosine, with the same effectiveness and side effects. The drawbacks of ATP also include chest pain, dyspnea, and sinus bradycardia up to AVB, which may require interruption of FFR measurement. A more convenient and safer hyperemic agent may facilitate the use of FFR measurement in the cardiac catheterization laboratory.

Nicorandil possesses a nitrate moiety and also can open ATP-sensitive potassium channels, thereby causing dilation of both macro- and micro-vascular coronary systems, and has been reported to be safe and have cardio-protective effects *via* intracoronary administration (12, 17–20). Some studies showed that the hyperemic efficacy of an IC bolus injection of nicorandil 2 mg was comparable with that of a continuous infusion of adenosine for FFR measurements (13, 21). While so far, there were no similar reports in Chinese patients, nor have there been any reports of native nicorandil used as hyperemia medication in FFR measurement, and also no reports of IC nicorandil compared with ATP. All these were the objectives of this research.

In other studies, there was no difference between hyperemic efficacies with nicorandil and adenosine, while in this study, FFR values with nicorandil 2 mg IC were commonly a little bit higher than those with ATP infusion (*p* < 0.001). Maybe it is partially because of a little bit higher dose of ATP infusion in this study. Adenosine or ATP is usually administered at 140 mcg/kg/min, and will be increased to 180 mcg/kg/min in some cases to obtain a more satisfying hyperemia effect. In this study, the ATP was given at a dose of 167 mcg/kg/min, the same dose as our routine clinical practice, 4 mg ATP dissolved in saline to make 40 ml ATP saline liquid, and was given at weight (kg) × 10 ml/h. This method saves the trouble of calculating the infusion speed (ml/h) according to different weights to obtain the 140–180 mcg/kg/min dose.

It is known that the use of ATP has the potential to induce transient AVB regardless of IV or IC administration. IV ATP infusion caused more prolongation of the PR interval than IC NIC administration (22). In this study, we found that transient AVB occurred in 2 (4.34%) patients with an infusion of ATP, and none with an IC bolus of nicorandil. This result was coherent with other recent studies. Takashima and Lim also concluded the incidence of AV block after ATP was significantly higher than nicorandil (*p* < 0.001). Additionally, Takashima noted significant (*p* < 0.001) fluctuations in FFR after ATP administration. This phenomenon may be explained by the different pharmacological mechanisms of nicorandil and ATP. A larger sample and multi-center studies are looking forward to perform and discover the principle.

In this study, a strong and linear correlation was observed between them with an infusion of ATP and an IC bolus of nicorandil 2 mg (*r* = 0.983, FFR_nicorandil_ = 0.937 × FFR_ATP_ + 0.061, *R*^2^ = 0.966). The two methods have a similarity of identifying functionally significant stenosis. If functionally significant stenosis was defined as FFR value ≤ 0.75, the consistency rate for IC nicorandil and infusion ATP was 96.08%; while if defined as FFR value ≤ 0.8, the consistency rate was 90.20%. In 5 lesions with the discrepancy, FFR values with ATP were all between 0.79 and 0.82. Therefore, the IC bolus of nicorandil can be used for FFR measurement.

In our study, we chose to inject nicorandil with an intracoronary bolus, which is similar to most of the studies in this field. More importantly, it is more accurate than an intravenous bolus of nicorandil in calculating FFR. First, it is more quickly with an IC bolus of nicorandil than that with an intravenous bolus and it reduces cyclic change in FFR. Second, in clinical practice, after bolus nicorandil, some saline is injected quickly to ensure all 2 mg of medication flow into the coronary artery. Third, the time to the lowest FFR with an IC bolus was shorter than with an intravenous bolus. It was published that IC nicorandil could reduce a cyclic change in FFR. The cyclic change in FFR was smaller after nicorandil-FFR than after ATP-FFR. Variation in adenosine concentration and an insufficient trough level of adenosine in the coronary artery may cause cyclic hyperemia. Some studies suggest that NIC may solve this phenomenon (23). Thus, physicians might find it easier to determine the FFR value during the procedure.

The advantages of the IC bolus of nicorandil as a hyperemia method in clinical practice include: (1) IC nicorandil bolus did not cause much discomfort; while nearly all infusions of ATP caused chest pain or dyspnea; (2) the influence on blood pressure, heart rate, and cardiac conduction was smaller with IC nicorandil than with ATP, and could be used in some patients with contraindications to ATP; (3) An IC bolus injection method is easy and simple, does not require an additional procedure for deep vein access; and (4) IC bolus nicorandil also has evidence of its benefit in patients with the slow flow or myocardial infarction.

### Limitations

First, multicenter studies with a larger population and with clinical endpoints are required to confirm the promising findings observed in this study. Second, we did not investigate the hyperemic efficacy of higher doses (>2 mg) of nicorandil. Third, as this was not a blinded study, there could have been a small amount of subjectivity in the interpretation of pressure tracings.

## Conclusion

This study suggests that an IC bolus injection of nicorandil is a simple, safe, and effective way to induce a stable hyperemia state for invasive physiological evaluation in patients undergoing angiography in a cardiac catheterization laboratory. The use of this novel agent may encourage interventional cardiologists to perform FFR measurements in their patients to optimize interventional procedures. While if FFR with nicorandil 2 mg lies between 0.79 and 0.82, it is better to reevaluate with ATP or adenosine.

## Data availability statement

The original contributions presented in this study are included in the article/supplementary material, further inquiries can be directed to the corresponding author.

## Ethics statement

The studies involving human participants were reviewed and approved by The First Medical Center, Chinese PLA General Hospital. The patients/participants provided their written informed consent to participate in this study.

## Author contributions

All authors listed have made a substantial, direct, and intellectual contribution to the work, and approved it for publication.

## Abbreviations

ASA: aspirin
ATP: adenosine 5′-triphosphate
AVB: atrioventricular block
CAD: coronary artery disease
CCBs: calcium-channel blockers
EQ: equalize
FFR: fractional flow reserve
IC: intracoronary
iFR: instantaneous wave-free ratio
i.v.: intravenous
LAD: left anterior descending artery
LCX: left circumflex
LM: left main
LVEF: left ventricular ejection fraction
PCI: percutaneous coronary intervention
QCA: quantitative coronary angiography
RAAS: renin-angiotensin-aldosterone system
RCA: right coronary artery
VAS: visual analog scale.

## References

[1] Pijls NH Schaarden burgh P Manoharan G Boersma E Bech JW van’t Veer M Percutaneous coronary intervention of functionally nonsignificant stenosis: 5-year follow-up of the DEFER study. *J Am Coll Cardiol.* (2007) 49:2105–11. 10.1016/j.jacc.2007.01.087 17531660

[2] Tonino PA De Bruyne B Pijls NH Siebert U Ikeno F van’ t Veer M Fractional flow reserve versus angiography for guiding percutaneous coronary intervention. *N Engl J Med.* (2009) 360:213–24. 10.1056/NEJMoa0807611 19144937

[3] De Bruyne B Pijls NH Kalesan B Barbato E Tonino PA Piroth Z Fractional flow reserve guided PCI versus medical therapy in stable coronary disease. *N Engl J Med.* (2012) 367:991–1001. 10.1056/NEJMoa1205361 22924638

[4] Park SH Koo BK. Clinical applicationsoffractional flow reserve in bifurcation lesions. *J Geriatr Cardiol.* (2012) 9:278–84. 10.3724/SP.J.1263.2012.05091 23097658 PMC3470027

[5] Kern MJ Lerman A Bech JW De Bruyne B Eeckhout E Fearon WF Physiological assessment of coronary artery disease in the cardiac catheterization laboratory: a scientific statement from the american heart association committee on diagnostic and interventional cardiac catheterization, council on clinical cardiology. *Circulation.* (2006) 114:1321–41. 10.1161/CIRCULATIONAHA.106.177276 16940193

[6] Wijns W Kolh P Danchin N Folliguet T Garg S Huber K Guidelines on myocardial revascularization. *Eur Heart J.* (2010) 31:2501–55. 20802248 10.1093/eurheartj/ehq277

[7] Patel MR Dehmer GJ Hirshfeld JW Patel MR Smith PK Chambers CE ACCF/SCAI/STS/AATS/AHA/ASNC/HFSA/SCCT 2012 Appropriate use criteria for coronary revascularization focused update: a report of the American College of Cardiology Foundation Appropriate Use Criteria Task Force, Society for Cardiovascular Angiography and Interventions, Society of Thoracic Surgeons, American Association for Thoracic Surgery, American Heart Association, American Society of Nuclear Cardiology, and the Society of Cardiovascular Computed Tomography. *J Am Coll Cardiol.* (2012) 59:857–81. 22296741 10.1016/j.jacc.2011.12.001

[8] Interventional Cardiology Group, Chinese Society of Cardiology, Chinese Medical Association, Thrombus Prevention Committee. Chinese guidelines for percutaneous coronary intervention. *Chin J Cardiovasc Dis.* (2016) 44:1–20.

[9] Khan ZA Akbar G Saeed W Malik S Khan F Sardar MR. Ventricular fibrillation with intracoronary adenosine during fractional flow reserve assessment. *Cardiovasc Revasc Med.* (2016) 17:487–9. 10.1016/j.carrev.2016.07.004 27477304

[10] Pijls NH Tonino PA. The crux of maximum hyperemia: the last remaining barrier for routine use of fractional flow reserve. *J Am Coll Cardiol Interv.* (2011) 4:1093–5. 10.1016/j.jcin.2011.08.007 22017934

[11] Kasumi I Fujii K Satoru O Hiroto T Rui I Shingo Y Influence of caffeine intake on intravenous adenosine-induced fractional flow reserve. *J Cardiol.* (2020) 76:472–8. 10.1016/j.jjcc.2020.05.011 32532583

[12] Markham A Plosker GL Goa KL. Nicorandil: an updated review of its use in ischaemic heart disease with emphasis on its cardio-protective effects. *Drugs.* (2000) 60:955–74. 10.2165/00003495-200060040-00007 11085202

[13] Jang HJ Koo BK Lee HS Park JB Kim JH Seo MK Safety and efficacy of a novel hyperaemic agent, intracoronary nicorandil, for invasive physiological assessments in the cardiac catheterization laboratory. *Eur Heart J.* (2013) 34:2055–62. 10.1093/eurheartj/eht040 23396491

[14] Pijls NH van Son JA Kirkeeide RL De Bruyne B Gould KL. Experimental basis of determining maximum coronary myocardial, and collateral blood flow by pressure measurements for assessing functional stenosis severity before and after PTCA. *Circulation.* (1993) 87:1354–67. 10.1161/01.cir.87.4.1354 8462157

[15] Sparv D Götberg M Harnek J Persson T Madsen Hardig B Erlinge D. Assessment of increasing intravenous adenosine dose in fractional flow reserve. *BMC Cardiovasc Disord.* (2017) 17:60. 10.1186/s12872-016-0463-4 28196527 PMC5310024

[16] Petraco R Escaned J Sen S Nijjer S Asrress KN Echavarria-Pinto M Classification performance of instantaneous wave-free ratio (iFR) and fractional flow reserve in a clinical population of intermediate coronary stenoses: results of the ADVISE registry. *Eur Interv.* (2013) 9:91–101. 10.4244/EIJV9I1A14 22917666

[17] Miyazawa A Ikari Y Tanabe K Nakajima H Aoki J Iijima R Intracoronary nicorandil prior to reperfusion in acute myocardial infarction. *Eur Interv.* (2006) 2:211–7. 19755263

[18] Lee HC An SG Choi JH Lee TK Kim J Kim JH Effect of intra-coronary nicorandil administration prior to reperfusion in acute ST segment elevation myocardial infarction. *Circ J.* (2008) 72:1425–9. 10.1253/circj.cj-08-0212 18724016

[19] Kim SJ Kim W Woo JS Ha SJ Kang WY Hwang SH Effect of myocardial protection of intracoronary adenosine and nicorandil injection in patients undergoing non-urgent percutaneous coronary intervention: a randomized controlled trial. *Int J Cardiol.* (2012) 158:88–92. 10.1016/j.ijcard.2011.01.011 21256606

[20] Kobatake R Sato T Fujiwara Y Sunami H Yoshioka R Ikeda T Comparison of the effects of nitroprusside versus nicorandil on the slow/no-reflow phenomenon during coronary interventions for acute myocardial infarction. *Heart Vessels.* (2011) 26:379–84. 10.1007/s00380-010-0065-5 21110199

[21] Jung HO Seung KB Kim PJ Ihm S Kang DH Youn HJ Comparison between nicorandil and adenosine in the measurement of coronary flow reserve using a Doppler guide wire. *Korean Circ J.* (2002) 32:391–7. 10.4070/kcj.2002.32.5.391

[22] Tanaka N Takahashi Y Ishihara H Kawakami T Ono H. Usefulness and safety of intracoronary administration of nicorandil for evaluating fractional flow reserve in Japanese patients. *Clin Cardiol.* (2015) 38:20–4. 10.1002/clc.22344 25626396 PMC6711086

[23] Takami H Sonoda S Muraoka Y Sanuki Y Kashiyama K Fukuda S Impact of additional intracoronary nicorandil administration during fractional flow reserve measurement with intravenous adenosine 50 -triphosphate infusion. *J Cardiol.* (2017) 69:119–24. 10.1016/j.jjcc.2016.01.018 26947100

